# Polymicrobial Diseases

**DOI:** 10.3201/eid0901.020487

**Published:** 2003-01

**Authors:** Harry W. Haverkos

**Affiliations:** Food And Drug Administration, Rockville, Maryland

Polymicrobial diseases involve multiple infectious agents and are referred to as complex, complicated, mixed, dual, secondary, synergistic, concurrent, polymicrobial, coinfections. This new book, a collection of 21 chapters written by a variety of authors, reviews mixed infections in animals and humans. The chapters are gathered into sections on polyviral diseases, polybacterial diseases, viral and bacterial infections, fungal infections, infections resulting from microbe-induced immunosuppression, and a concluding perspective. Polymicrobial diseases described include abscesses, AIDS-related opportunistic infections, conjunctivitis, gastroenteritis, hepatitis, multiple sclerosis, otitis media, periodontal diseases, respiratory diseases, and genital infections. Approximately two-thirds of the chapters deal with human diseases; the others discuss infections in cattle, goats, and pigs.

The chapters are generally well written with a focus on microbiology, pathogenesis, and to a lesser degree, treatment. The chapters on abscesses, multiple sclerosis, and mixed mycotic infections are especially informative. The chapter on abscesses provides a comprehensive review of the microbiology processes involved, the role of anaerobes in mixed infections, and animal models. The section on viruses and multiple sclerosis is provocative in its proposal that several viruses might coexist and interact to promote multiple sclerosis and other neurologic diseases. The list of candidate etiologic agents includes *Human herpesvirus*-6, human T-lymphotropic virus type 1, measles viruses, JC virus, Epstein-Barr virus, and herpes simplex virus-1. The chapter on mixed mycotic infections adequately discusses how fungi interact by mechanisms such as commensalism, opportunism, mixed colonization, co-isolation, and dual and polymicrobial infection.

Growing two or more microbes in the laboratory in a clinical situation does not prove that a polymicrobial infection is the cause of the disease. The editors and authors do not provide a framework similar to that of Robert Koch or Bradford Hill, which one can use to decipher the role(s) of each candidate agent in a polymicrobial infection. An limited discussion is provided on the role of noninfectious factors, such as genetics of the host, retained “hardware”, alcohol in hepatitis, or tobacco use in respiratory diseases. How each of the chapters was selected for inclusion and what other topics were considered is not clear.

The reference lists are one of the book’s strengths but also a weakness. The lists are extensive, occupying about 30% of the book’s pages. Prioritizing the outside readings on each topic would have been useful. Several of the chapters might have been combined, such as the two on periodontal diseases, those on retroviruses, and those on respiratory diseases in humans, cattle, and pigs. In the next edition, the authors might explore the polymicrobial etiology of Reye syndrome, autoimmune disorders, atherosclerosis, and cancers, such as Kaposi sarcoma, hepatocellular sarcoma, and cervical cancer. I recommend the book to those who think beyond the “single agent, single disease” framework and imagine multifactorial causes for those diseases currently listed as “etiology unknown.”

